# Human native lipoprotein-induced *de novo *DNA methylation is associated with repression of inflammatory genes in THP-1 macrophages

**DOI:** 10.1186/1471-2164-12-582

**Published:** 2011-11-25

**Authors:** Rubén Rangel-Salazar, Marie Wickström-Lindholm, Carlos A Aguilar-Salinas, Yolanda Alvarado-Caudillo, Kristina BV Døssing, Manel Esteller, Emmanuel Labourier, Gertrud Lund, Finn C Nielsen, Dalia Rodríguez-Ríos, Martha O Solís-Martínez, Katarzyna Wrobel, Kazimierz Wrobel, Silvio Zaina

**Affiliations:** 1 Department of Medical Sciences, University of Guanajuato, León, Mexico; 2 Experimental Cardiovascular Research Laboratory, Lund University, Malmö, Sweden; 3 National Institute for Medical Sciences and Nutrition "S. Zubirán", Mexico City, Mexico; 4 Cancer Epigenetics and Biology Program, IDIBELL, Barcelona, Spain; 5 Center for Genomic Medicine, Rigshospitalet, Copenhagen, Denmark; 6 Asuragen Inc., Austin, Texas; 7 Department of Genetic Engineering, CINVESTAV, Irapuato, Mexico; 8 Department of Chemistry, University of Guanajuato, Guanajuato, Mexico

## Abstract

**Background:**

We previously showed that a VLDL- and LDL-rich mix of human native lipoproteins induces a set of repressive epigenetic marks, *i.e. de novo *DNA methylation, histone 4 hypoacetylation and histone 4 lysine 20 (H4K20) hypermethylation in THP-1 macrophages. Here, we: 1) ask what gene expression changes accompany these epigenetic responses; 2) test the involvement of candidate factors mediating the latter. We exploited genome expression arrays to identify target genes for lipoprotein-induced silencing, in addition to RNAi and expression studies to test the involvement of candidate mediating factors. The study was conducted in human THP-1 macrophages.

**Results:**

Native lipoprotein-induced *de novo *DNA methylation was associated with a general repression of various critical genes for macrophage function, including pro-inflammatory genes. Lipoproteins showed differential effects on epigenetic marks, as *de novo *DNA methylation was induced by VLDL and to a lesser extent by LDL, but not by HDL, and VLDL induced H4K20 hypermethylation, while HDL caused H4 deacetylation. The analysis of candidate factors mediating VLDL-induced DNA hypermethylation revealed that this response was: 1) surprisingly, mediated exclusively by the canonical maintenance DNA methyltransferase DNMT1, and 2) independent of the Dicer/micro-RNA pathway.

**Conclusions:**

Our work provides novel insights into epigenetic gene regulation by native lipoproteins. Furthermore, we provide an example of DNMT1 acting as a *de novo *DNA methyltransferase independently of canonical *de novo *enzymes, and show proof of principle that *de novo *DNA methylation can occur independently of a functional Dicer/micro-RNA pathway in mammals.

## Background

Atherosclerosis is characterised by the accumulation of lipids, extracellular matrix, smooth muscle, inflammatory and immune cells in the arterial wall [1]. Diet-related and environment-related factors are pivotal determinants of atherosclerosis risk, thus epigenome remodelling by such factors has been proposed as an important underlying molecular mechanism for that disease [2]. According to this view, environmental and nutritional risk factors might impose stable epigenetic "hits" during an individual's lifetime that, possibly in synergy with other concomitant molecular changes, cause anti- or pro-atherogenic gene expression patterns [3,4]. Indeed, altered DNA methylation patterns have been detected in atherosclerosis [5-7]. Such changes may at least in part be caused by abnormal lipoprotein profiles, given their central role in atherogenesis [1]. This idea is supported by our previous observation that a very low density- and low density lipoprotein (VLDL and LDL, respectively)-rich lipoprotein mix (VLR) induces global *de novo *DNA methylation in THP-1 human macrophages, in addition to other epigenetic modifications associated with non-permissive chromatin. The latter include loss of histone 4 acetylation and an increase in histone 4 lysine 20 hypermethylation [7]. Furthermore, work by other groups showed that oxidised LDL (oxLDL) modulates promoter methylation of the estrogen receptor alpha and matrix metallopeptidase-2 and -9 genes in vascular smooth muscle cells [8,9].

The present work analysed the effects of lipoprotein-induced *de novo *DNA methylation on gene expression in THP-1 macrophages. Furthermore, it tested the involvement of individual DNA methyltransferase enzymes and known DNA methylation-mediating pathways. Our findings are discussed in the context of the current knowledge on the role of native lipoproteins in epigenetic gene regulation and inflammation.

## Methods

### Cell culture, lipoprotein isolation

THP-1 monocytes were differentiated to macrophages as previously described [7]. For Oil Red O staining and intracellular lipid determination, macrophages were processed as described [7]. Human VLDL, LDL and high-density lipoprotein (HDL) were isolated and mixed to create the VLR mix (concentrations in μg protein/ml: 68 VLDL, 32.1 LDL, 91.1 HDL) that was used to stimulate THP-1 macrophages in serum-free conditions for 24 h as previously described [7]. The rationale for VLR composition is outlined in [7] and in brief is the following: 1) relative lipoprotein proportions reproduce a hyperlipidaemic profile similar to the one observed in APOE-null mice and in diabetic patients; 2) final absolute lipoprotein concentrations are ~10-fold lower than hyperlipidaemic levels to avoid cell toxicity; 3) triglyceride-rich lipoprotein levels in VLR were sufficient to induce intracellular lipid (Oil Red O-stained) droplets in our conditions (not shown) and increased intracellular triglyceride levels (additional file 1: Figure S1), suggesting that THP-1 macrophages exposed to VLR represented a model of lipid-loaded counterparts observed in hyperlipidaemic atherosclerosis [1]. Each lipoprotein preparation represented a pool of a variable number of donors with unspecified lipidaemic status, obtained either in Malmö, Sweden (4 independent preparations) or Mexico City, Mexico (3 independent preparations), as specified in the Results section for each experiment. Lipoprotein preparations were stored at -80°C for less than 6 months and used within 3 days of thawing.

### Genome expression arrays

Affymetrix GeneChip Human Genome U133 Plus 2.0 Arrays were hybridized with labelled total RNA extracted by using the RNeasy system (Qiagen), scanned with an Affymetrix GeneChip Scanner 3000 according to standard protocols at the microarray facility, Rigshospitalet, Copenhagen, Denmark. RNA integrity was checked by agarose electrophoresis at the source laboratory and again at the microarray facility. The dChip software (build April 15, 2005) was used for normalization and modelling using the PM-only model. Array data were deposited in the GEO database (http://www.ncbi.nlm.nih.gov/geo/) with accession numbers GSE9101 and GSM230349-GSM230360. For pathway analysis, the BioCarta (http://www.biocarta.com) and Reactome (http://www.reactome.org) databases were searched with the modeled-based gene set analysis (MGSA) that analyzes all categories at once by embedding them in a Bayesian network in which gene response is modelled as a function of the activation of the categories [10,11]. Probabilistic inference is used to identify the active categories. Pathways were selected if identified by both databases and with p < 0.05.

### Gene expression

For quantitative RT-PCR, one-tenth of cDNA obtained from 1 μg total RNA was amplified by using the LightCycler Fast Start DNA MasterPLUS SYBR green I system (Roche) according to manufacturer's instructions in a LightCycler 1.5 (Roche). Primer sequences were obtained with qPrimerDepot (http://primerdepot.nci.nih.gov). Relative expression levels were calculated by subtracting the average *GAPDH *CP from the CP of the target gene obtained from the same cDNA and applying the formula 2^-ΔCP^. CP values were calculated by the LightCycler 1.5 software. All samples were in triplicate. The primers used are listed in additional file 2: Table S1. DNMT1 and -2 expression was analysed by RT-PCR with the following oligonucleotides: 5'-AAGTAGAAGCGGTTGGGGCCG-3' and 5'-GGCAGGCCCAATGAGACTGAC-3' (*DNMT1*); 5'-ATGGAGCCCCTGCGGGTGCTG-3' and 5'-GTGAATGGCTGGCAGGGAGGG-3' (*DNMT2*). *GAPDH *cDNA was used as loading control by using commercial oligonucleotides (Clontech, Mountain View, CA). Immunoblotting was performed using the antibody Abcam no. ab14601 (Dicer) and the following Santa Cruz Biotechnology products: sc-271729 (DNMT1), sc-365001 (DNMT2), sc-20703 (DNMT3A), sc-20704 (DNMT3B), sc-20705 (DNMT3L), sc-7210 (α-actin). Positive controls were ES-2 cell lysate (Santa Cruz Biotechnology) for DNMT3L and human Ficoll gradient-purified peripheral blood mononuclear cells for other DNMTs and Dicer. ELISA kits were used to detect IL-6 (R&D Systems, Abingdon, UK, cat. D6050) and IL-23 p19 (eBioscience, San Diego, CA, cat. 88-7234) in cell supernatants according to manufacturer's instructions.

### Global DNA methylation and histone posttranslational modifications

Global DNA methylation, histone acetylation and methylation levels were measured as described [7,12].

### Gene-specific DNA methylation

For analysis of bisulfite-treated DNA, 1 μg DNA was modified with the EZ DNA Methylation-Gold™ system (Zymo Research) according to manufacturer's instructions. One-tenth of modified DNA was amplified with gene-specific primers listed in additional file 2: Table S1. PCR products were either subjected to melting analysis using LightCycler Fast Start DNA Master^PLUS ^SYBR green I in a Light Cycler 1.5 machine (Roche) [13] or sequenced (minimum 10 clones per studied sequence). For combined bisulfite restriction analysis (COBRA), bisulfite-modified DNA was digested with 1U TaqI or BstUI/μg initial DNA and amplified with gene-specific primers in the same Light Cycler system. For relative quantification, the average mock sample CP was subtracted from the CP of enzyme-treated triplicate samples and the formula 2^-ΔCP ^was used. CP values were calculated by the LightCycler 1.5 software. All samples were in triplicate.

### Proteomics

For two-dimensional electrophoresis of nuclear proteins, nuclei were purified essentially as described in Schreiber et al. [14]. Protein electrophoresis was performed according to standard protocols with first dimension separation performed in a pH3-10 range. Spots were counted in silver-stained gels by using an UMAX scanner and the ImageMaster™ 2D Platinum software (Amersham Biosciences, Little Chalfont, UK) after correcting for background according to manufacturer's instructions. For kinomics studies, an immunoblot-based Kinetworks™ Phospho Site Screen (KPSS-4.1, Kinexus, Vancouver, Canada) was employed. The manufacturer performed blotting, immunodetection and data normalisation.

### DNMT knockdown

At 48 h of differentiation, cells were transfected with 20 μM siRNA or carrier (controls) and siPORT Amine (both from Ambion) according to manufacturer's instructions for a 6-well plate format, in serum-free medium for 24 h, followed by a change to complete medium. At the end of differentiation, cells were stimulated with VLDL for 24 h and collected by gentle scraping. Total siRNA incubation thus amounted to 72 h. To validate our transfection protocol, we performed pilot experiments with a FAM-labelled siRNA (Ambion ID no. AM4622). Two *DNMT1*-specific (Ambion ID no. 110915 and 110917, respectively) and one *DNMT2*-specific (Ambion ID no. 111450) pre-designed siRNAs were used. Cell viability was measured by trypan blue staining.

### Micro-RNA (miRNA) array

Total RNA was isolated and fractionated using methods optimized for small RNA recovery and the resulting miRNA fraction labelled and hybridized onto two colour microarrays as described [15].

## Results

### Down-regulation of gene expression in VLR-stimulated cells

**Figure 1:**
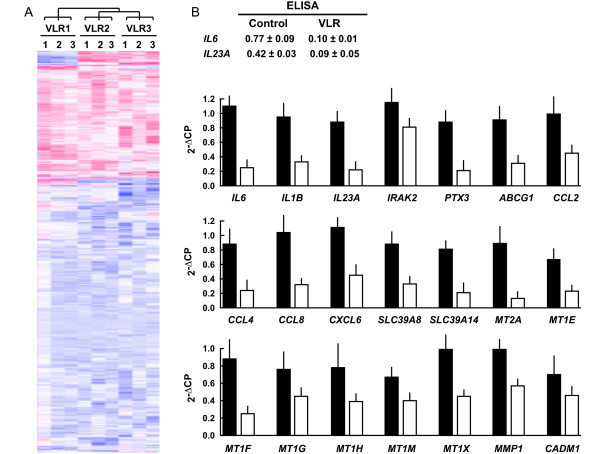
**Effects of VLR on gene expression**. A, all-gene expression clustering in VLR-stimulated THP-1 macrophages compared to controls - *i.e*. no VLR - following a 24 h-challenge. VLR1-VLR3, individual lipoprotein preparations, each in triplicate. Blue and red, relatively down- and up-regulated genes, respectively. Clustering shows consistent differences between control and VLR-stimulated cells. Notice the excess of VLR-down-regulated genes compared to up-regulated ones. B, validation of array gene expression data by ELISA for IL-6 and IL23 p19 (indicated with gene symbol *IL23A *for simplicity; data are indicated as ng/ml/10^6 ^cells) and by qRT-PCR (graphs below ELISA values). Genes are ordered according to the functional grouping criteria of Table 1. qRT-PCR y-axis values are 2^-ΔCP ^in comparison with the average of the *GAPDH *CP triplicates. Black bars, control; white bars, VLR-stimulated. Bars, standard deviations. P < 0.05 in all cases.

Our previous data showed that VLR causes global DNA hypermethylation [7]. VLR-induced *de novo *DNA methylation might target a combination of gene promoters and gene body sequences, presumably causing mainly transcriptional repression in the former case and sustained or enhanced transcription in the latter [16,17]. Reasoning that a general transcriptional repression should be observed if promoters are a major target of VLR-induced *de novo *DNA methylation, we conducted a genome expression array analysis (Affymetrix GeneChip Human Genome U133 Plus 2.0 array) to compare control and VLR-stimulated cells. In this and all experiments indicated below, stimulations were carried out for 24 h. Three independent lipoprotein preparations obtained in Sweden were analysed, each in triplicate. Counting triplicate controls, the experiment amounted to 12 individual arrays. Clustering analysis of all genes showed an excess of down-regulated transcripts compared to up-regulated ones in VLR-stimulated cells, and consistence across lipoprotein preparations (Figure 1A). All subsequent gene expression analyses presented here were conducted on transcripts showing a > 3-fold change in expression with a P < 0.001 (ANOVA test) in all three lipoprotein preparations compared to controls. Genes that were down-regulated by VLR were 3.2-fold more abundant than up-regulated ones (70 vs. 22, respectively) (additional file 3: Table S2). The sum of differences in RNA expression between VLR-stimulated and control cells averaged for the three lipoprotein preparations was 47,335.8 and 8,435.1 for down- and up-regulated genes, respectively, representing a 5.6-fold excess of transcripts down-regulated by VLR compared to up-regulated ones.

Interestingly, a serendipitous finding suggested that corresponding effects were detectable at the proteome level. As part of a parallel project assessing the impact of VLR on nuclear protein expression, we performed two-dimensional electrophoresis of nuclear proteins in two independent experiments using one lipoprotein preparation obtained in Sweden and independent from the ones used for expression arrays. The results revealed a 24-44% reduction in total spot count in VLR-treated compared to control cells (1,723 vs. 2,258 and 1,339 vs. 2,409 spots in the two experiments; data not shown). These observations, although limited to an incomplete sample of the whole genome, are consistent with the idea that a global decrease in gene expression is expected to result in a decrease in translation.

### Identity and independent validation of genes down-regulated by VLR

Pro-inflammatory genes (*IL1B*, *IL6*, *IL7R*, *IL23A*), genes participating in inflammatory responses (*IRAK2, PTX3, TRAF1*) [18-21] and cholesterol transport (*ABCG1*) were pronouncedly down-regulated by VLR (Table 1; gene symbols will be used throughout the text, for corresponding gene names see additional file 3: Table S2). *IL23A *and *IL1B *RNA showed a > 20-fold repression and ranked first and third, respectively, among down-regulated genes. Other down-regulated genes included members of chemokine, metallothionein and Zn/cation transporter families. By contrast, genes up-regulated by VLR were a more heterogeneous group. VLR did not alter the expression of any cell cycle- or apoptosis related genes, in accordance with our earlier biochemical and cellular morphology data (additional file 3: Table S2) [7]. Accordingly, pathway analysis according to the BioCarta and Reactome databases consistently indicated that chemotaxis (CCR5-mediated, enrichment score (ES) = 0.47, p = 0.011), inflammation (IL1R-mediated, ES = 0.30, p = 0.006) and Zinc transport (solute carrier 39 (SLC39) family-mediated, ES = 0.14, p = 0.005) pathways were significantly down-regulated in VLR-stimulated cells compared to controls.

**Table 1:** Characteristics of genes down-regulated by VLR.

Function	Gene symbol
Inflammation	IL1B, IL6, IL7R, IL23A, IRAK2, PTX3, TRAF1
Cholesterol transport	ABCG1
Chemotaxis	CCL2, CCL3, CCL4, CCL8, CCL20, CXCL6, CCR7
Solute carrier family (Zn, cation transport)	SLC7A2, SLC39A8, SLC39A14
Metal binding, control of oxidative stress gb:AL031602 (MT1E-like)	MT2A, MT1E, MT1F, MT1G, MT1H, MT1M, MT1X,
Matrix metalloproteinase	MMP1
Cell adhesion	CADM1

For array data validation, cells were stimulated with a pool of 3 independent lipoprotein preparations obtained in Mexico. Array expression data were confirmed by ELISA (IL-6, IL-23 p19) and qRT-PCR (the latter 2 genes and 19 additional genes) (Figure 1B). We used *GAPDH *as reference transcript as its expression was not affect by VLR, as assessed by whole genome expression array data (not shown). All genes showed a significant (*P *< 0.05) decrease in expression. As control we used *BTF3*, randomly chosen among genes whose expression levels were not significantly changed by VLR in either expression microarrays or validation qRT-PCR (not shown).

### Promoter methylation status of genes down-regulated by VLR

**Figure 2:**
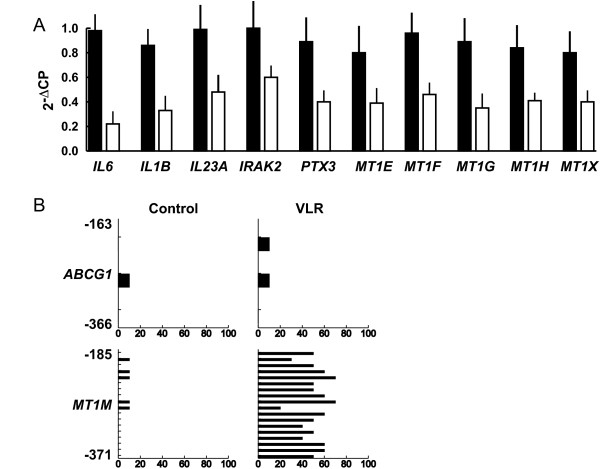
**Methylation analysis of VLR-regulated genes**. A and B, COBRA and bisulfite-modified DNA sequence analysis, respectively. A, values on the y-axis are 2^-ΔCP ^in comparison with the average of mock digestion triplicates. An increase in methylation results in relatively low 2^-ΔCP ^values. Black bars, control; white bars, VLR-stimulated. Bars, standard deviations. B, numbers on the vertical axis indicate the position of the analyzed sequence relative to the transcription start site (bp). Ticks represent CpG dinucleotides. Horizontal bars represent the extent of methylation of the corresponding residues in the clones analyzed.

The general transcriptional repression induced by VLR suggested that promoters are major targets for *de novo *DNA methylation by this lipoprotein mixture. Thus, selected expression-validated genes belonging to relevant functional groups (inflammation, cholesterol transport, metallothioneins, see Table 1; 12 genes total) were first screened for promoter methylation status by melting analysis. DNA extracted from an aliquot of the same cells used for gene expression validation (see previous paragraph) was used. Results showed hypermethylation in VLR-treated cells compared to controls in all cases but for the cholesterol transporter *ABCG1 *and the control gene *BTF3 *(not shown). The same genes were subsequently analyzed by COBRA (10 genes) or bisulfite modified DNA sequencing (2 genes) to assess whether that increase in methylation was significant. Our COBRA analysis can detect significant changes in DNA methylation, although it cannot yield absolute levels. All genes but *ABCG1 *showed significant (*P *< 0.05) promoter hypermethylation in VLR-treated cells compared to controls (Figure 2A,B).

Gene mapping revealed that 41 of the 70 down-regulated genes (or ~59%) were found in clusters on chromosomes, *i.e*. in groups of 3 or more genes per chromosome arm (additional file 4: Table S3). This gene distribution was significantly different from a random one (*P *< 0.00003, Chi-square test). The expected number of genes per chromosome in a random distribution was calculated correcting for chromosome size http://www.ncbi.nlm.nih.gov/guide/genomes-maps/. In one case, 7 down-regulated metallothionein genes clustered in a 1 Mb portion of 16q. Another noticeable example was the down-regulation of a group of chemokine and Zn/cation transporter genes belonging to 3 distinct clusters in 4q, 8p and 17q. Furthermore, the inflammatory genes *IL6, IL1B *and *PTX3 *were included in clusters (additional file 4: Table S3). No such clustering was observed among up-regulated genes.

### Effects of individual lipoproteins on de novo DNA methylation and histone modifications

**Figure 3:**
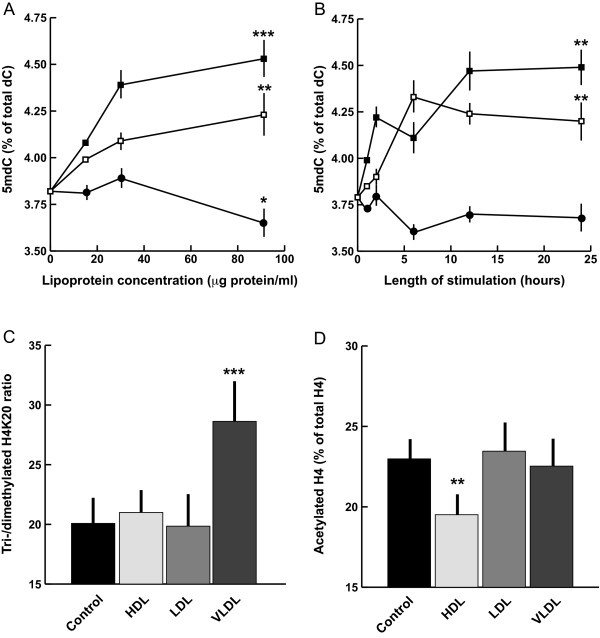
**Epigenetic responses to individual lipoproteins**. A, dose-response for global DNA methylation. Solid squares, VLDL; open squares, LDL; solid circles, HDL. 5 mdC, 5-methyldeoxycytosine. For simplicity, significance levels are shown only for highest dose-responses compared to respective controls. B, time-course of global DNA methylation response following stimulation with individual lipoprotein at 91.1 μg protein/ml concentration. Symbols as in A. C and D, effects on histone posttranslational modifications. Significance levels are for comparisons with the respective control. One, two and three stars indicate P < 0.05, P < 0.01 and P < 0.005 significance level, respectively (Scheffé test). Bars, standard deviations.

In order to assess which lipoprotein species of VLR are most active in inducing *de novo *DNA methylation and histone modifications, we stimulated cells with increasing amounts of each individual lipoprotein up to the highest dose used in VLR - *i.e*. 91.1 μg protein/ml HDL - for 24 h. One of the three lipoprotein preparations obtained in Mexico were used. Both VLDL and LDL induced a significant *de novo *DNA methylation with relative potency VLDL > LDL, or a 17% and 10% increase in DNA methylation, respectively, compared to control (Figure 3A). Noticeably, the effect of HDL if any, was a marginal DNA hypomethylation. The response to HDL was not due to a biphasic time-course, since global DNA methylation did not vary across the 0-24 h. interval in macrophages challenged with each lipoprotein at 91.1 μg protein/ml concentration (Figure 3B). Histone post-translational modification analysis revealed a distinct specificity in the responses to lipoproteins. VLDL only induced an increase in H4K20 hypermethylation, whereas HDL only caused a loss of acetylated H4 (40% increase and 15% decrease, respectively, compared to control cells) (Figure 3C,D). Neither histone modification was significantly affected in LDL-stimulated cells.

### DNMT1 mediates VLDL-induced de novo global and gene promoter DNA methylation

Mammalian DNA methylation is performed by a family of DNA methyltransferases (DNMTs) of which the best characterised are DNMT1, -2, -3A, -3B and -3L. DNMT1 is considered to be the canonical maintenance DNMT, *i.e*. the enzyme that copies pre-existing DNA methylation patterns during replication, whereas *de novo *DNMT activity is largely attributed to DNMT3A and -3B [22,23]. On the other hand, DNMT3L is enzymatically inactive and cooperates with DNMT3A to direct DNA methylation of sequences containing specific histone post-translational modifications, while RNA methylation is probably a distinctive biological activity of DNMT2 [24-26].

**Figure 4:**
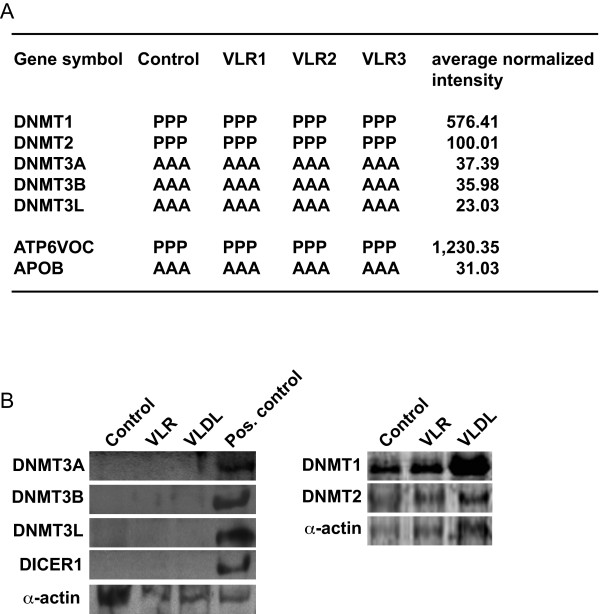
**Expression of DNMTs and DICER1 in THP-1 macrophages**. A, Affymetrix present/absent calls (P and A, respectively) and average normalized probeset intensity are shown for arrays (grouped in triplicates) addressing the effects of VLR. *ATP6VOC *and *APOB *are included as examples of a highly expressed and silent gene, respectively. VLR1 through VLR3, independent VLR preparations. B, Immunoblot analysis of DNMT and DICER1 expression. VLDL was used at 91.1 μg protein/ml concentration. Representative alpha-actin immunoreactivity using peripheral blood mononuclear cells as positive control is shown.

**Figure 5:**
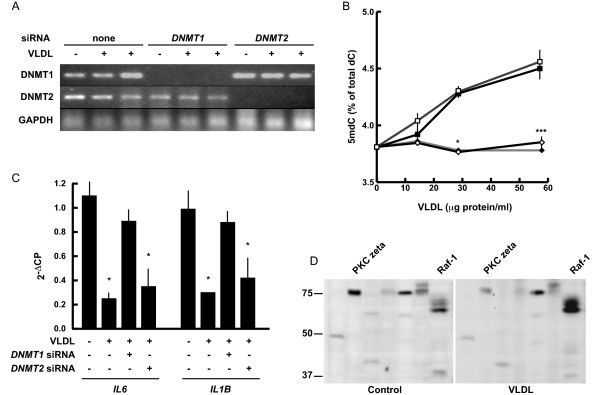
**DNMT1 mediates VLDL-induced de novo DNA methylation**. A, RT-PCR analysis of DNMT expression in siRNA-treated cells stimulated with two preparations of VLDL at 58 μg protein/ml. B, effects of siRNA-mediated knockdown of DNMT1 or DNMT2 on total 5 mdC content. Solid squares, control; open squares, *DNMT2*-specific siRNA; solid diamonds, *DNMT1*-specific siRNA ID no. 110915; open diamonds, *DNMT1*-specific siRNA ID no. 110917 (both from Ambion). Asterisks indicate statistically significant comparisons between siRNA-treated and control cells. For simplicity, when cells treated with either *DNMT1*-specific siRNA show different significance levels, only the lower one is shown (*, P < 0.05; ***, P < 0.001). C, effects of siRNA treatment on the methylation status of *IL6 *and *IL1B*, as assessed by COBRA. DNAs from the two *DNMT1*-specific siRNAs were pooled for this analysis. Statistical significance is indicated as in B. Bars, standard deviations. D, VLR alters baseline phosphorylation of PKC zeta and Raf-1. Fields of Kinexus Kinetworks™ Phospho Site Screen KPSS-4.1 containing PKC zeta T410 and Raf-1 S259 in control and VLR-stimulated cells. Raf-1 migrates as multiple bands reflecting multiple phosphorylation states [55]. Positions of relevant MW standard are shown on the left (kDa).

The four independent sets of genome expression arrays each conducted in triplicate for a total of 12 individual arrays presented in the whole genome expression array experiment (see above) consistently indicated that THP-1 macrophages express DNMT1 and -2, but not DNMT3A, DNMT3B or DNMT3L, irrespective of whether stimulated with VLR or unstimulated (Figure 4A). The presence of DNMT1 and -2, and absence of DNMT3A and -B were confirmed by RT-PCR (using the same pool of lipoproteins prepared in Sweden for the microarray experiment; not shown) and immunoblot (using the same pool of material produced in Mexico as used for microarray validation; Figure 4B). The apparent increase in DNMT1 expression in VLR-stimulated cells did not exceed 25% compared to control, when normalized by α-actin levels. Therefore, expression data suggested that either DNMT1 or DNMT2 or both mediate VLDL -induced *de novo *DNA methylation in THP-1 macrophages. To assess the relative contribution of DNMT1 and -2 in VLDL-induced *de novo *DNA methylation in THP-1 cells, we employed an RNAi-mediated knockdown approach. One of the lipoprotein preparations obtained in Mexico, different from the one used to assess responses to individual lipoproteins (see above) was used in this case. siRNA treatment lowered DNMT expression to undetectable levels in our RT-PCR conditions (Figure 5A). Two distinct *DNMT1*-specific siRNAs abolished VLDL-induced *de novo *DNA methylation and promoter hypermethylation of the VLR-downregulated genes *IL6 *and *IL1B*, whereas a DNMT2-specific siRNA had no significant effect (Figure 5B,C). DNMT1 knockdown did not result in any change in cell viability compared to controls (not shown) in contrast with previous data obtained in HeLa cells, thus suggesting that the effects of DNMT1 knock-down are cell type-specific [27]. We attempted to complement the RNAi approach with re-expression of a siRNA-insensitive *DNMT1 *RNA, but pilot transient expression experiments using lipofectamine and an empty vector (pcDNA, Invitrogen) failed, likely due to the inability of THP-1 macrophage to survive multiple transfections during differentiation. Furthermore, to explore possible signalling pathways mediating DNMT1 activation by VLDL, we employed an immunoblot-based kinomics analysis of the phosphorylation status of 45 kinase substrates. These experiments were conducted with the lipoprotein preparation obtained in Sweden and used in DNMT RT-PCR experiments (see above, this paragraph). Results were representative of a pool of three independent cell lysates in each analysed group. Following normalization and considering only > 2-fold differences, VLDL produces small changes, as it decreased protein kinase C zeta (PKCzeta) T410 phosphorylation by 3.4-fold, and marginally increased baseline v-raf-1 murine leukemia viral oncogene homolog 1 (Raf-1) S259 phosphorylation (2-fold; Figure 5D). Since T410 phosphorylation is an activating modification of PKCzeta, our preliminary data suggest that VLDL decreases PKCzeta signalling, whether by inhibiting phosphorylation and/or expression of that signalling factor [28].

### Involvement of miRNAs and Dicer

Among factors guiding DNMTs to their targets, the Dicer and micro-RNA (miRNA) pathway seems to play a pivotal role at least in some cell types, as genetic evidence shows that Dicer is necessary to maintain the non-permissive state of selected genomic regions at least in some cell types in mammals [29,30]. The RNase Dicer mediates the maturation of miRNAs, a class of endogenous small non-coding RNAs known to control gene expression at the post-transcriptional level. In addition to the latter function, miRNAs and other Dicer substrates direct DNA methylation to specific sequences in various organisms including mammals [31,32].

To explore the hypothesis that VLDL causes *de novo *DNA methylation by inducing the expression of specific miRNAs or other Dicer substrates capable to direct DNMT1 to specific targets, we undertook a miRNA expression analysis. In addition, we examined the expression levels of Dicer in THP-1 macrophages. A pool of 2 of the lipoprotein preparations obtained in Sweden and used in the whole genome microarray experiment was used. miRNA expression microarrays showed that VLDL had marginal effects on miRNA expression levels, as following array global centered normalization, no significant miRNA differential expression - *i.e*. > 2-fold - was detected in two independent biological replicates. As for Dicer expression, whole genome expression arrays yielded mixed indications, as only 3 of the 5 *DICER1 *probes were scored as "present". Nonetheless, RT-PCR (not shown) and immunoblotting (conducted with distinct cellular and lipoprotein material in Scandinavia and Mexico; Figure 4B) revealed that Dicer (*DICER1 *gene) was not expressed in lipoprotein-stimulated or control THP-1 macrophages. Therefore, our data provide evidence that VLDL-induced *de novo *DNA methylation is not a Dicer-mediated process in THP-1 macrophages.

## Discussion

The present work shows that VLR, a human native VLDL- and LDL-rich lipoprotein mix, induces a net global decrease in gene expression that mirrors the previously reported global *de novo *DNA methylation caused by VLR in THP-1 macrophages [7]. Proteome data independently support these findings, although the effects of VLR were more prominent on protein species than on transcripts. This observation points to VLR affecting a combination of translation repression, post-translational modification regulation and nuclear protein stability. VLR-induced transcript down-regulation includes pathways involved in fundamental macrophage functions such as inflammation, chemotaxis, metal and cation transport. The down-regulation of pro-inflammatory genes is a prominent effect of VLR. Seven pro-inflammatory genes are repressed, in addition to one solute carrier gene (*SLC7A2*) that participates in macrophage activation by various cytokines [33]. Although apparently in contrast with data showing atherogenic effects of VLDL, these distinct effects of VLR are consistent with existing literature indicating that stimulation by native VLDL *per se *results in a weak, if any, inflammatory response in cell culture models [34]. For example, VLDL induces inflammatory marker expression only in synergy with cytokines in human aortic endothelial cells and macrophages [35-37]. Similar results were obtained in a side-by-side gene expression analysis in THP-1 macrophages stimulated with oxidised LDL, or oxidised or native chylomicrons remnants. Oxidised LDL and oxidised chylomicrons produced a radically different gene expression pattern from the one induced by native chylomicrons, including a down-regulation of *ABCA1 *by the latter, resembling the effects of VLR on *ABCG1 *presented here [9,38]. Furthermore, native LDL is a poor activator of the NLRP3 inflammasome, a protein complex involved in IL-1beta production, compared with corresponding oxidised versions [39]. Based on our results and supported by evidence discussed above, we conclude that VLR-induced *de novo *DNA methylation in THP-1 macrophages does not reflect a pro-atherogenic response as our earlier data seemed to indicate [7], rather it underlies an anti-inflammatory response. It is possible that in normal physiological conditions circulating native VLDL and possibly other lipoproteins buffer macrophage functions by limiting pro-inflammatory gene activity. The observation that exogenous lipoproteins can protect against endotoxin-induced death and decrease plasma TNF *in vivo *provides ground to this idea [40]. On the other hand, when pro-inflammatory signals are present presumably above a critical threshold, particularly in association with lipoprotein oxidation, or act chronically as in sustained hyperlipidaemia, anti-inflammatory gene regulation mechanisms would be overrun. As a consequence, native lipoproteins cease to exert negative controls on macrophage function and indeed act as an activating switch by synergising with inflammatory factors. Persson and co-workers discussed this basic idea in detail [37].

For all analyzed genes but *ABCG1*, VLR-induced silencing is associated with *de novo *DNA methylation at the corresponding promoter. Interestingly, the non-random distribution of down-regulated genes suggests that VLR regulates gene expression by a coordinated mechanism resembling instructive epigenetic regulation reported in cancer [41].

In addition, our results are in apparent contrast with published data suggesting that DNA hypermethylation may underlie a pro-inflammatory response in some conditions [42]. Our data complement the latter observations, by showing that DNA hypermethylation is not invariably associated with inflammation. Rather, we suggest that specific epigenetic modifications are imposed on distinct sets of sequences in a stimulus-specific fashion thus inducing specific gene expression patterns, although the resulting global epigenetic parameters - *i.e*. total genome DNA methylation level - may be similar.

Furthermore, we observed a potentially interesting selectivity in *de novo *DNA methylation and H4K20 hypermethylation responses between individual lipoprotein species. Our data indicate that the response to VLR must be the sum of activities of factors specific for or present at different amounts in each lipoprotein species. The mechanisms underlying the observed differential effects on histone post-translational modifications are currently unknown and deserve investigation. Clearly, understanding the mechanisms by which lipoproteins modulate chromatin structure in macrophages and other cell types requires a detailed screening of lipoprotein components. The observation that lipoprotein preparations from Sweden or Mexico produced consistent effects in whole genome expression array analysis and its validation, suggests the preliminary conclusion that the factor eliciting the responses described here is a structural lipoprotein component probably not qualitatively or quantitatively affected by diet. Interestingly, recent evidence shows that palmitic acid, an abundant pro-inflammatory fatty acid of endogenous and dietary origin, promotes global DNA hypermethylation in primary human myocytes [43].

As for the identity of intracellular factors mediating VLDL-induced *de novo *DNA methylation, the absence of the canonical *de novo *DNMTs DNMT3A and -3B was unexpected in THP-1 macrophages. Our data indicate that DNMT1 is necessary and sufficient for *de novo *DNA methylation in response to VLDL, in contrast with its widely accepted role as canonical maintenance DNMT. Interestingly, these observations are supported by our preliminary kinome data showing that VLDL lowers the cellular levels of T410-phosphorylated - *i.e*. active - PKCzeta, which has been recently shown to inhibit DNMT1 [44]. Previous studies demonstrated that DNMT1 might participate in *de novo *DNA methylation in cooperation with DNMT3A and -3B [45,46]. Accordingly, exogenous DNMT1 expression induced *de novo *methylation of a relatively small number of genes in HEK-293T cells, possibly in cooperation with endogenous DNMT3A/3B [47]. To our knowledge, the only previous example of an independent *de novo *methylation activity for DNMT1 is the demonstration that this enzyme re-establishes somatic patterns of non-CpG methylation following their erasure in the germline [48]. The present study provides new evidence that DNMT1 can perform *de novo *DNA methylation independently of maintenance DNMTs. A *de novo *activity for DNMT1 may be physiologically relevant in tissues in which an age-related decline of DNMT3A/B expression has been documented [49,50]. Interestingly, DNMT1 might be a specific mediator of *de novo *DNA methylation in response to pro-inflammatory signals, as IL-6 induces upregulation and nuclear translocation of DNMT1 [51-53]. As for DNMT2, the absence of any effects of this DNMT on DNA methylation confirms previous literature data [25].

In addition, our data provide genetic evidence on the involvement of the Dicer pathway in epigenetic responses to VLDL. Taken together, the absence of Dicer expression and the lack of any effect of VLDL on miRNA production demonstrate that the latter factors or other small non-coding RNAs generated by Dicer do not mediate VLDL-induced *de novo *DNA methylation in THP-1 macrophages. These observations indicate that chromatin regulation by Dicer-mediated pathways if present in mammals, is confined to specific cell types rather than being a universal mechanism. Nonetheless, it is possible that Dicer-independent RNA-mediated DNA methylation operates in THP-1 cells as reported in plants [54].

## Conclusions

We provide insights into gene targets and potential mechanisms for native lipoprotein-induced epigenetic gene regulation in THP-1 macrophages. These findings contribute to understanding interactions between the genome and lipids or other lipoproteins components in health and disease. Furthermore, by studying candidate factors involved in epigenetic responses to lipoproteins, we provide new evidence that the canonical maintenance DNMT1 is capable of *de novo *DNA methylation activity, and show proof of principle that the Dicer pathway is not indispensable for *de novo *DNA methylation in all human cells.

## Authors' contributions

RRS carried out DNMT1 knock-down experiments and cell culture. MWL performed cell culture work and lipoprotein purification, and contributed to experimental design. CAAS provided donors for and purified human lipoproteins and revised the manuscript critically. YAC performed immunoblots and DNA work. KBVD carried out proteomics and DNMT expression assays. ME provided important intellectual contributions. EL carried out the miRNA array analysis. GL substantially contributed to experimental design and revised the manuscript critically. FCN provided important intellectual contributions. DRR performed cloning. MOSM performed qPCR. KW and KW performed methylcytosine quantification. SZ designed the study, carried out all experimental work not contributed for by other authors and wrote the initial manuscript draft. All authors read and approved the manuscript.

## Supplementary Material

Additional file 1 **VLR increases intracellular lipids in THP-1 macrophages**. Quantification of intracellular triglycerides and cholesterol in VLR-stimulated THP-1 macrophages. Black and grey bars, triglycerides and cholesterol, respectively. ***, P < 0.005. Data are presented as average and s.d. of triplicates of one experiment. Click here for file

Additional file 2 **Primers used in the study**. List of gene-specific primers used for RT-PCR and DNA methylation analysis. Click here for file

Additional file 3 **Genes significantly regulated by VLR**. Genes are ordered in descending order of expression change (VLR-stimulated vs. control cells, left column). Notice the excess of negatively regulated genes. Gene counts differ from the ones indicated in the text, as some transcripts are represented on arrays more than once. Click here for file

Additional file 4 **Clustering of genes down-regulated by VLR**. Chromosomal position and cluster size for genes that are down-regulated by VLR. Click here for file
