# Supplementary material for: Human native lipoprotein-induced de novo DNA methylation is associated with repression of inflammatory genes in THP-1 macrophages
Source: BMC Genomics. 2011 Nov 25;12:582. doi: 10.1186/1471-2164-12-582 (PMC3247910; doi:10.1186/1471-2164-12-582)
Supplement: Additional file 2 — Primers used in the study. List of gene-specific primers used for RT-PCR and DNA methylation analysis. [file 1471-2164-12-582-S2.PDF]

Additional file 2: table S1 – Primers used for qRT-PCR and DNA methylation analyses.

Gene symbol	qRT-PCR	DNA methylation
ABCG1	GGCCACCAACTCACCACTAT	GATTTGGAGGAATTTTAGTTT[B]
	CAGGGACCTTTCCTATTCGG	CTAAAAAACAATTCTATTCCC
BTF3	TTCTTATCGTCTGCTGTGGC	GGTTAAGATTATAAAAGGTTG[C(B)]
	ATGAACCAGGAGAAACTCGC	CAAAAAAATTAACTCCTAAATACC
CADM1	CAACCTCTCCCTCGATCACT	
	GGCTTCTGCTGTTGCTCTTC	
CCL2	AGGTGACTGGGGCATTGAT	
	GCCTCCAGCATGAAAGTCTC	
CCL4	GCTTGCTTCTTTTGGTTTGG	
	CTTTTCTTACACCGCGAGGA	
CCL8	AAAGCAGCAGGTGATTGGAA	
	AGATGAAGGTTTCTGCAGCG	
CXCL6	GGCAATTTTATGATGCATGG	
	TTTGTCTGGACCCGGAAG	
IL1B	CCTGAAGCCCTTGCTGTAGT	GTAGTTTGTTGTGTTTTGTGTTT[C(T)]
	AGCTGATGGCCCTAAACAGA	CCCCTTTCCTTTAACTTAATTAT
IL6	GTCAGGGGTGGTTATTGCAT	GGTAGGGTAGTAGTTAATTTTT[C(B)]
	AGTGAGGAACAAGCCAGAGC	ATTCTTCTATATTCTAACTCTCC
IL23A	CCACACTGGATATGGGGAAC	AGGATTAGTTAGAGTTATTGATAG[C(T)]
	CTCAGTGCCAGCAGCTTTC	TTAACTAAAAACCACCTAAC
IRAK2	TGGAATGGGACACCTGATTT	TTTATTTAGGTTGGAGTATAAGG[C(T)]
	GCAACTTGTGGACCTCCTGT	TCCCAATACTTTAAAAAACC
MMP1	TTGTGGCCAGAAAACAGAAA	
	TTCGGGGAGAAGTGATGTTC	
MT1E	CGCACTCACTCTTCTTGCAG	GAGGATTTGGATAAATGTGTTT[C(B)]
	AATGGACCCCAACTGCTCTT	AAATTACCTTCCACCCTCCT
MT1F	TGCATTTGCACTCTTTGCAC	YGAAGGTTAGGATTTTTAGGTAT[C(B)]
	AGTCCAGTCTCTCCTCGGCT	CTAAATAAACTATATACAACAA
MT1G	GCATTTGCACTCTTTGCACT	TTGTTTTTATAGTTAGGAGA[C(B)]
	GGAACTCTAGTCTCGCCTCG	ATTACCCTCRACTCCAAAAAAAA
MT1H	AGGAGCAGCAGCTCTTCTTG	GGGATTTTAGGAAAGTTTTA[C(B)]
	CAATGGACCCCAACTGCT	TAAACTACTTATTTCACCCTAC
MT1M	AGGAGCAGCAGCTCTTCTTG	TGGAAGGTAAAGGTAATTTT[B]
	CAACTGCTCCTGCACCACT	AACTAAACTAAACTAAACCCAAC
MT1X	CTTTGCAGATGCAGCCCT	AGAGGGAGAGGTAGGTAATG[C(B)]
	GCAAATGCAAAGAGTGCAAA	CTTTATAATCCRAAAAAAAAACC
MT2A	TCTTTGCATTTGCAGGAACC	
	CAACCTGTCCCGACTCTAGC	
PTX3	ATCTTCTTGGAACGCATTGG	AATTTTAGTAATTTGGGAGG[C(T)]
	GGTGCTAGAGGAGCTGCG	CACCACACCCAATTAATTTTTAT
SLC39A8	GGCCCCTTCAAACAGGTACA	
	TGCTGTCACAGAAGCTAATGG	
SLC39A14	GCAGCTTCATGGTGACTGAA	
	GCTAAGCTGCTTCTGCCG	

Abbreviations: [B], bisulfite-modified DNA sequencing. [C(B)] and [C(T)], COBRA using *Bst*UI or *Taq*I, respectively.
