# Supplementary material for: Human native lipoprotein-induced de novo DNA methylation is associated with repression of inflammatory genes in THP-1 macrophages
Source: BMC Genomics. 2011 Nov 25;12:582. doi: 10.1186/1471-2164-12-582 (PMC3247910; doi:10.1186/1471-2164-12-582)
Supplement: Additional file 3 — Genes significantly regulated by VLR. Genes are ordered in descending order of expression change (VLR-stimulated vs. control cells, left column). Notice the excess of negatively regulated genes. Gene counts differ from the ones indicated in the text, as some transcripts are represented on arrays more than once. [file 1471-2164-12-582-S3.PDF]

Additional file 3: table S2 - Genes significantly regulated by VLR. Genes are ordered in descending order of expression change (VLR-stimulated vs. control cells, left column). Notice the excess of negatively regulated genes. Gene counts differ from the ones indicated in the text, as some transcripts are represented on arrays more than once.

Fold change, VLR-stimulated vs.control	Gene symbol	Gene name	Accession number
**6,31**	PDK4	pyruvate dehydrogenase kinase 4	AV707102
**4,62**	RNASE6	ribonuclease, RNase A family, k6	NM_005615
**4,55**	MNDA	myeloid cell nuclear differentiation antigen	NM_002432
**4,38**	FABP4	fatty acid binding protein 4, adipocyte	NM_001442
**4,12**	CD300A	CD300A antigen	AF020314
**4,06**	FLJ12442	hypothetical protein FLJ12442	NM_022908
**3,99**	FLJ22811	Homo sapiens cDNA: FLJ22811, clone KAIA2944	N93191
**3,95**	MGC24133	hypothetical protein MGC24133	AW662189
**3,9**	FPR1	formyl peptide receptor 1	NM_002029
**3,89**	MAP3K5	mitogen-activated protein kinase kinase kinase 5	AW500340
**3,8**	FAM13A	family with sequence similarity 13, member A1	NM_014883
**3,67**	FAM13A	family with sequence similarity 13, member A1	AK027138
**3,63**	MTUS1	mitochondrial tumor suppressor 1	AL096842
**3,49**	CNR1	cannabinoid receptor 1 (brain)	U73304
**3,45**	ITGA4	integrin, alpha 4 (antigen CD49D, alpha 4 subunit of VLA-4 receptor)	BG532690
**3,43**	ST14	suppression of tumorigenicity 14 (colon carcinoma, matriptase, epithin)	U20428
**3,41**	TREM2	triggering receptor expressed on myeloid cells 2	NM_018965
**3,2**	FUCA1	fucosidase, alpha-L- 1, tissue	NM_000147
**3,19**	CD52	CD52 antigen (CAMPATH-1 antigen)	N90866
**3,14**	ST14	suppression of tumorigenicity 14 (colon carcinoma, matriptase, epithin)	NM_021978
**3,08**	IPCEF1	phosphoinositide-binding protein PIP3-E	AW166711
**3,07**	LMO2	LIM domain only 2 (rhombotin-like 1)	NM_005574
**3,06**	ITGA4	integrin, alpha 4 (antigen CD49D, alpha 4 subunit of VLA-4 receptor)	NM_000885
**3**	LMO2	similar to CG32736-PA	BF573638
**-3,02**	PBEF1	pre-B-cell colony enhancing factor 1	BF575514
**-3,03**	SLC39A8	solute carrier family 39 (zinc transporter), member 8	NM_022154
**-3,05**	CPM	carboxypeptidase M	BE878495
**-3,09**	SOCS3	suppressor of cytokine signaling 3	AI244908
**-3,1**	RNF144B	IBR domain containing 2	AI953847

**-3,11**	SLC39A14	solute carrier family 39 (zinc transporter), member 14	D31887
**-3,11**	PAICS	phosphoribosylaminoimidazole carboxylase, phosphoribosylaminoimidazole succinocarboxamide synthetase	NM_006452
**-3,13**	GPD2	glycerol-3-phosphate dehydrogenase 2 (mitochondrial)	AA613031
**-3,14**	CTH	cystathionase (cystathionine gamma-lyase)	AL354872
**-3,15**	NCF1	neutrophil cytosolic factor 1 (47kDa, chronic granulomatous disease, autosomal 1)	AW072388
**-3,15**	NRP2	neuropilin 2	AK024680
**-3,16**	IL1RN	interleukin 1 receptor antagonist	U65590
**-3,19**	NCF1	neutrophil cytosolic factor 1 (47kDa, chronic granulomatous disease, autosomal 1)	NM_000265
**-3,23**	TP53INP2	tumor protein p53 inducible nuclear protein 2	AL109824
**-3,25**	LOC285628	hypothetical protein LOC285628	AL389942
**-3,26**	KTELC1	hypothetical protein MGC4308	BC006475
**-3,27**	MCOLN2	mucolipin 2	AY083533
**-3,29**	PTX3	pentraxin-related gene, rapidly induced by IL-1 beta	NM_002852
**-3,32**	FLJ23749	hypothetical protein FLJ23749	BF680438
**-3,36**	LOC389185	hypothetical LOC389185	AI733564
**-3,37**	QPCT	glutaminyl-peptide cyclotransferase (glutaminyl cyclase)	NM_012413
**-3,44**	BHLHE41	basic helix-loop-helix domain containing, class B, 3	BE857425
**-3,5**	KIAA1533	KIAA1533	AB040966
**-3,5**	IL1RN	interleukin 1 receptor antagonist	BE563442
**-3,51**	MT2A	metallothionein 2A (MT2A), mRNA	NM_005953
**-3,56**	MT1G	metallothionein 1G	NM_005950
**-3,6**	MCOLN2	mucolipin 2	AV713773
**-3,62**	MT1E	metallothionein 1E (functional)	BF217861
**-3,62**	IL1RN	interleukin 1 receptor antagonist	AW083357
**-3,64**	CCL8	chemokine (C-C motif) ligand 8	AI984980
**-3,65**	SEMA3C	sema domain, immunoglobulin domain (Ig), short basic domain, secreted, (semaphorin) 3C	NM_006379
**-3,65**	LOC727730	metallothionein 1H-like	AF333388
**-3,69**	MT1X	metallothionein 1X	NM_002450
**-3,74**	MT1H	metallothionein 1H	NM_005951
**-3,77**	MT1F	metallothionein 1F (functional)	M10943
**-3,78**	MT1X	metallothionein 1X	NM_005952
**-3,83**	IER3	immediate early response 3	NM_003897
**-3,93**	DUSP5	dual specificity phosphatase 5	U16996

**-3,97**		Human DNA sequence from clone RP5-1174N9 on chromosome 1p34.1-35.3.	AL031602
**-3,98**	CLEC4E	C-type lectin domain family 4, member E	BC000715
**-4,05**	MST4	Mst3 and SOK1-related kinase	NM_016542
**-4,15**	PARM1	prostate androgen-regulated mucin-like protein 1 (DKFZP564O0823 protein)	AI659927
**-4,17**	UPP1	uridine phosphorylase 1	NM_003364
**-4,2**	CD82	Kangai 1 (suppression of tumorigenicity 6, prostate; CD82 antigen)	AI870617
**-4,27**	CADM1	immunoglobulin superfamily, member 4D	AA640422
**-4,3**	MT1F	metallothionein 1F (functional)	BF246115
**-4,34**	CXCL6	chemokine (C-X-C motif) ligand 6 (granulocyte chemotactic protein 2)	NM_002993
**-4,48**	SERPINE2	serine (or cysteine) proteinase inhibitor, clade E (nexin, plasminogen activator inhibitor type 1), member 2	AL541302
**-4,59**	NM_018000	likely ortholog of mouse dilute suppressor	NM_018000
**-4,66**	TRAF1	TNF receptor-associated factor 1	NM_005658
**-4,74**	CD80	CD80 antigen (CD28 antigen ligand 1, B7-1 antigen)	BC042665
**-4,79**		Homo sapiens hqp0376 protein	AF078844
**-4,81**	GCH1	GTP cyclohydrolase 1 (dopa-responsive dystonia)	NM_000161
**-4,84**	TARP	T cell receptor gamma variable 9	M27331
**-4,9**	IGSF6	immunoglobulin superfamily, member 6	NM_005849
**-5,15**	IRAK2	interleukin-1 receptor-associated kinase 2	AI246590
**-5,59**	CCL2	chemokine (C-C motif) ligand 2	S69738
**-6,66**	PKIG	protein kinase (cAMP-dependent, catalytic) inhibitor gamma	NM_007066
**-6,91**	CCL4	chemokine (C-C motif) ligand 4	NM_002984
**-6,93**	LAMP3	lysosomal-associated membrane protein 3	NM_014398
**-7**	CCL20	chemokine (C-C motif) ligand 20	NM_004591
**-7,19**	MMT1	matrix metalloproteinase 1 (interstitial collagenase)	NM_002421
**-7,31**	IL7R	interleukin 7 receptor	BE217880
**-7,41**	C3orf1	chromosome 3 open reading frame 1	AA503803
**-7,47**	CCL3	chemokine (C-C motif) ligand 3	NM_002983
**-7,59**	IL7R	interleukin 7 receptor	NM_002185
**-7,73**	RASGRP1	RAS guanyl releasing protein 1 (calcium and DAG-regulated)	NM_005739
**-8,14**	IL6	interleukin 6 (interferon, beta 2)	NM_000600
**-8,76**	SLC7A2	solute carrier family 7 (cationic amino acid transporter, y+ system), member 2	AA876372
**-9,05**		Homo sapiens, clone IMAGE:4794726	BF513121
**-11,31**	CCR7	chemokine (C-C motif) receptor 7	NM_001838

**-11,34**	ABCG1	ATP-binding cassette, sub-family G (WHITE), member 1	NM_004915
**-11,96**	C15orf21	chromosome 15 open reading frame 21	AI928428
**-12,91**	INDO	indoleamine-pyrrole 2,3 dioxygenase	M34455
**-19,29**	IL1B	interleukin 1, beta	M15330
**-19,66**	IL1B	interleukin 1, beta	NM_000576
**-19,67**	MT1K	metallothionein 1K	R06655
**-25,8**	IL23A	interleukin 23, alpha subunit p19	NM_016584
