# Supplementary material for: Human native lipoprotein-induced de novo DNA methylation is associated with repression of inflammatory genes in THP-1 macrophages
Source: BMC Genomics. 2011 Nov 25;12:582. doi: 10.1186/1471-2164-12-582 (PMC3247910; doi:10.1186/1471-2164-12-582)
Supplement: Additional file 4 — Clustering of genes down-regulated by VLR. Chromosomal position and cluster size for genes that are down-regulated by VLR. [file 1471-2164-12-582-S4.PDF]

Additional file 4: table S3 - Clustering of genes down-regulated by VLR.

Map	Gene symbol	Cluster size (Mb)
1p	*CTH,* gb:AL031602 (*MT1E*-like), *MCOLN2*	52
2q	*CCL20, GPD2, IL1B, IL1RN,* NM_018000, *NRP2*	43
3q	*C3orf1, CD80,* hypothetical LOC389185*, KTELC1, LAMP3, PTX3*	64
4q	*CXCL6, DKFZP564O0823, SLC39A8*	27
7p	*IL6, TARP, UPP1*	15
7q	*NCF1, PBEF1, SEMA3C, SERPINE2*	5
8p	*INDO, SLC7A2, SLC39A14*	15
16q	*MT1E, MT1F, MT1G, MT1H, MT1K, MT1X, MT2A*	1
17q	*CCL2, CCL3, CCL4, CCL8, CCR7, SOCS3*	6
